# An intelligent framework for advancing large-scale omics data integration

**DOI:** 10.1016/j.isci.2026.115928

**Published:** 2026-04-29

**Authors:** Mintian Cui, Shixi Wang, Fan Yang, Yifei Wang, Fanyu Kong, Ni Kong, Mengying Li, Xiaoyue Qiao, Zhen Xu, Ziyu Yan, Yu Yan, Jiamo Zhang, Kun Chen

**Affiliations:** 1 State Key Laboratory of Cardiovascular Diseases and Medical Innovation Center, Shanghai East Hospital, School of Life Sciences and Technology, Tongji University, Shanghai 200127, China; 2 Department of Internal Emergency Medicine and Critical Care, Shanghai East Hospital, Tongji University School of Medicine, Shanghai 200120, China; 3 Shanghai Key Laboratory of Signaling and Disease Research, Frontier Science Center for Stem Cell Research, School of Life Sciences and Technology, Tongji University, Shanghai 200092, China

**Keywords:** Bioinformatics, Computational bioinformatics, Transcriptomics

## Abstract

Clinical and biological insights from large-scale omics data are often limited by technical variability and analytical complexity. Here, we present BioinAI, a comprehensive framework that integrates an intelligent system with two algorithms, DeepAdvancer and stNiche, to enable effective data integration. Specifically, DeepAdvancer leverages a class-aware adversarial autoencoder to reconstruct gene expression profiles. When applied to 49,738 samples across 1,016 datasets, it uncovered a transcriptomic continuum and differential trajectory axes, which link diverse diseases through shared immune responses and distinct fate determinants. In the spatial context, stNiche leverages graph networks and symmetry-aware matching to identify functional cellular niches across heterogeneous slides. For instance, it identified a fibroblast-immune niche surrounding hair follicles in healthy skin that is lost in pathological states. BioinAI also provides an online conversational analysis platform, powered by multiple semi-agents, facilitating biological insight extraction from transcriptomic data.

## Highlights

DeepAdvancer reconstructs cross-platform transcriptomic expression profiles
• stNiche identifies functional spatial niches associated with disease
• BioinAI-Web enables conversational and automated transcriptomic data analysis
• Skin diseases share common and distinct molecular features

## Graphical abstract

**Figure:**
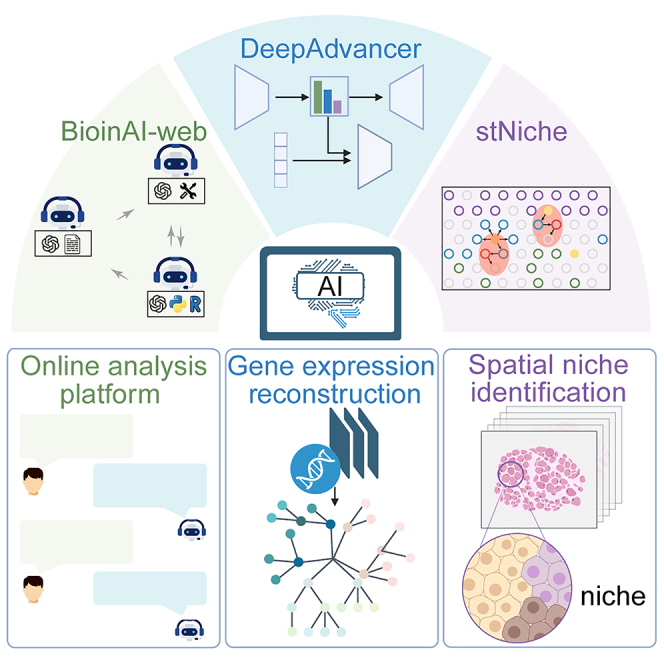

## Introduction

Gene expression regulation is a complex network, involving many molecular mechanisms and biological functions. Large-scale gene expression profiling has become a key resource for advancing our understanding of human diseases. An increasing number of studies aim to gain comprehensive biological insights through integrative analyses of various datasets.^1^^,^^2^ Conventional methods for bulk transcriptomic data integration, such as ComBat^3^ and limma,^4^ are commonly used strategies for removing technical variation. However, these methods, which are typically applied to datasets from the same platform, show a significant decline in performance when applied to cross-platform data analysis.^5^ In addition, integrative analysis of large-scale omics data are often labor-intensive, time-consuming, and require substantial expertise. Consequently, vast amounts of omics data remain underutilized. This highlights an urgent need for approaches to process and integrate large-scale, heterogeneous omics resources.

In addition to variations in gene expression levels, spatial context is also an essential dimension for understanding organ development and disease progression.^6^ Therefore, integrating single-cell and spatial transcriptomic data reveals cellular spatial distributions, offering deeper insights into tissue microenvironments. Under physiological and pathological conditions, the spatial distribution of cells in the tissue microenvironment often changes at the level of functional units. Importantly, these functional units play vital roles in biological processes such as immune surveillance and immune responses.^7^ Currently, approaches for identifying microenvironmental niches mainly rely on similarity-based clustering, based on the assumption that a niche is a region with homogeneous gene expression within individual samples.^8^^,^^9^ However, this assumption limits their ability to detect niches composed of heterogeneous cell populations across samples.

Recent advances in artificial intelligence (AI) and machine learning are transforming the landscape of large-scale integrative analyses,^10^ offering new approaches to analyze and interpret complex biological data. Intelligent agent systems based on large language models (LLMs) can autonomously perform tasks without constant human intervention by leveraging sequential reasoning, planning, and memory retention.^11^ Specifically, such systems comprise several key modules, including a perception module to receive input, a memory module to retain historical information, a planning module to develop strategies, an execution module to carry out plans, and a communication module to facilitate inter-agent dialogue. Recently, such systems have been applied to bioinformatics analysis. For example, CellAgent is an intelligent agent system designed for autonomously processing and analyzing single-cell RNA sequencing (scRNA-seq) datasets.^12^ However, current agent systems continue to suffer from stability and accuracy issues in data analysis, limiting their applicability in rigorous scientific research.^11^

To address these challenges, we developed BioinAI, a comprehensive bioinformatics framework comprising an online platform and two algorithms, DeepAdvancer and stNiche. DeepAdvancer employs a class-aware adversarial autoencoder to enable efficient integration of cross-platform gene expression data, minimizing technical noise while preserving critical biological information. For the integration of single-cell and spatial omics data, stNiche leverages spatial graph networks and symmetry-aware matching to identify spatial niches composed of diverse cell types, and further elucidates their functional roles and intercellular communication patterns. In addition, BioinAI integrates advanced LLMs to support omics data analysis, enhancing the depth and efficiency of transcriptomic research. To improve analytical stability and accuracy, we designed the semi-agent system, in which agents are equipped with predefined pipelines, core codebases, and clearly defined operational roles. This structure strengthens their task-specific reliability and interpretability. Overall, BioinAI provides an automated framework for transcriptomic analysis and offers useful tools for advancing the understanding of disease mechanisms.

## Results

### Design concept of BioinAI

**Figure 1:**
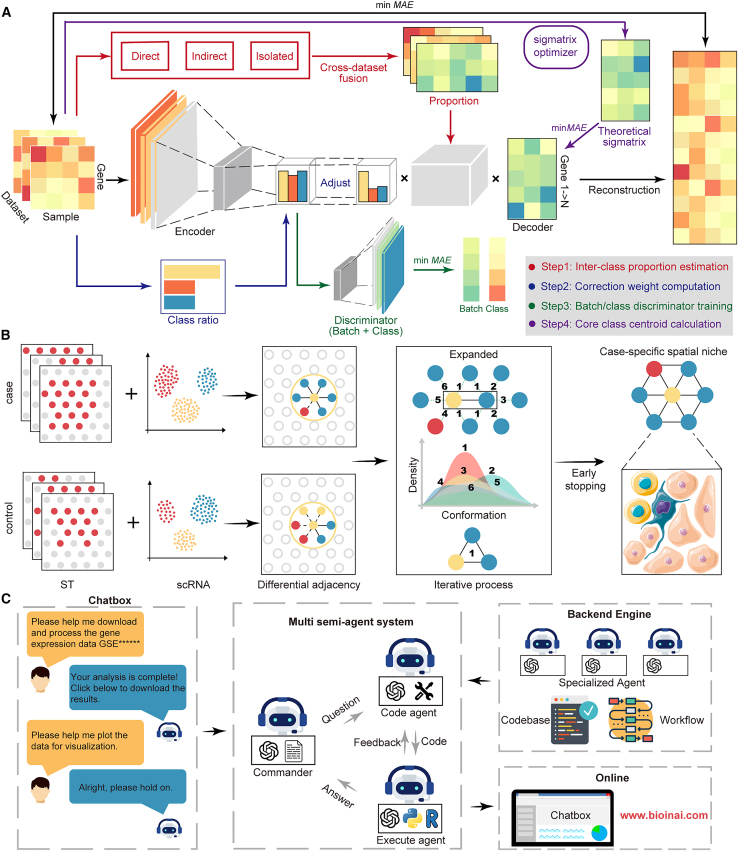
Schematic of the BioinAI Framework (A) The DeepAdvancer module integrates and reconstructs bulk transcriptomics datasets across different platforms. It first classifies disease relationships as direct, indirect, or isolated, guiding inter-class proportion calculations. An encoder then extracts features, dynamically adjusted by calculated class proportions. Competitive learning discriminators for class identity and batch identity are applied to remove batch effects and retain biological meaning. Additionally, expression values for foundational classes are optimized from the original datasets through iterative refinement. At last, a distinct optimization ensures minimal discrepancy between reconstructed and source data, thus preserving original biological signatures. (B) stNiche identifies biologically relevant cell niches composed of mixed cell types using spatial transcriptomics data. Niches are progressively learned by identifying the conformations of disease-specific cell spots. (C) A multiple semi-agents conversational system supports user-friendly and stable analysis of transcriptomics data via natural language interaction. The platform is accessible online at https://www.bioinai.com.

To enable effective integration of large-scale omics data across platforms, we developed DeepAdvancer, which is designed to reconstruct the biologically meaningful gene expression profiles. The decoder-derived weights were collapsed into a gene-level signal matrix. An additional signal-matrix loss was introduced to align this matrix with the expected signal matrix estimated during pre-training. Class-specific proportional terms derived from precomputed differential expression statistics were used to modulate class contributions. In addition, reconstruction loss is jointly optimized with dataset and class discriminator losses (Figure 1A). Collectively, these designs support the preservation of intrinsic biological signals while effectively mitigating batch effects.

Next, to enable integrative analysis of single-cell and spatial transcriptomic data across slides, we designed stNiche, which identifies functional niches composed of heterogeneous cell types within tissue contexts. Unlike conventional spatial domain identification methods,^8^^,^^9^ which primarily detect locally coherent regions within individual tissue sections, stNiche is tailored to capture niche structures that vary across biological conditions. Candidate niches are systematically constructed by evaluating structural variations between diseased and healthy tissues. By identifying spatial configurations significantly distinct within these states, the method can isolate niche-level patterns indicative of altered tissue organization. To address the presence of chiral or mirrored spatial structures, the framework incorporates an iterative expansion strategy and symmetry-aware matching. Furthermore, combining cell-type annotations enhances the biological interpretability of detected niches, enabling insights into cell-cell interactions and tissue microenvironments that are context-specific and spatially coherent (Figure 1B).

Lastly, we developed an online platform that enables intuitive and code-free omics analysis through interactive conversational interfaces. To mitigate hallucinations in LLMs, we introduced a semi-agent system. This framework combines automated AI-powered assistance with expert analytical scripts and standardized procedural control to improve the consistency and reproducibility of analyses. Specifically, the platform offers automated omics workflows, covering data acquisition, preprocessing, cell type annotation, differential expression analysis, enrichment analysis, and niche analysis (Figure 1C). In summary, BioinAI offers a comprehensive bioinformatics framework for omics data.

### DeepAdvancer reconstructs gene expression across 132 skin conditions

**Figure 2:**
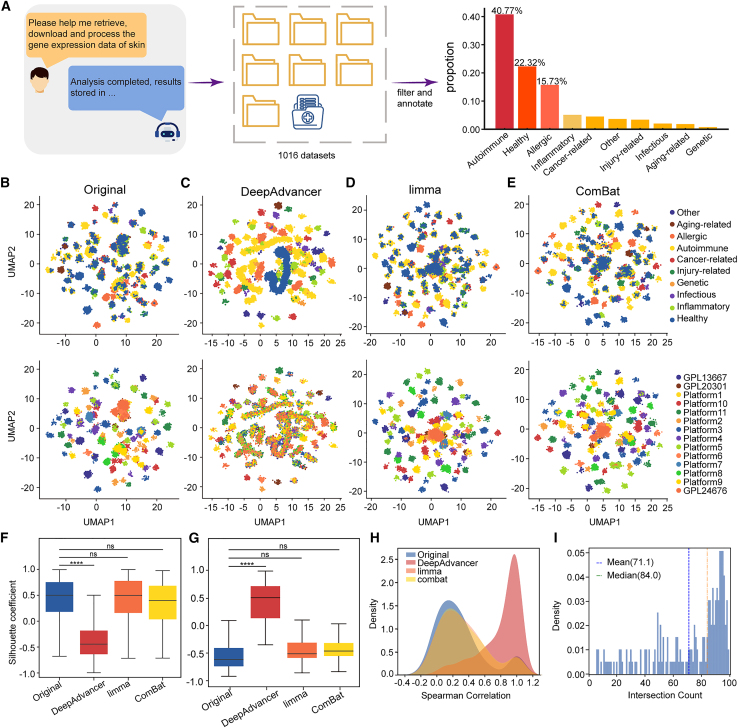
Comparison of batch correction methods on large-scale skin datasets (A) Data acquisition and processing were carried out through our BioinAI online platform. The right panel shows the proportion of annotated disease categories. (B–E) Uniform manifold approximation and projection (UMAP) visualization of the datasets before (B) and after batch correction using DeepAdvancer (C), limma (D), and ComBat (E). The upper row is colored by disease category, and the lower row is colored by dataset platform. (F and G) Silhouette coefficients by disease class (F) and dataset origin (G) for each method. Center lines indicate the median; box limits indicate the upper and lower quartiles; whiskers indicate the range. Statistical significance was assessed by Student’s *t* test. ∗∗∗∗*p* < 0.0001; ns, not significant. (H) Distribution of Spearman correlation for each method. (I) Histogram showing the intersection count of the top 100 genes ranked by expression fold-change between DeepAdvancer-corrected and reference data.

We began by evaluating the performance of DeepAdvancer in integrating large-scale omics datasets with substantial biological and technical heterogeneity. To this end, we retrieved a collection of gene expression profile datasets related to cutaneous pathologies. Leveraging the automated pipeline developed within the BioinAI platform, we systematically retrieved, annotated, and preprocessed a total of 49,738 samples across 1,016 datasets (Table S1). These datasets subsequently underwent rigorous filtering and annotation to facilitate downstream visualization and interpretation. The annotated categories included: healthy, autoimmune, cancer-related, infectious, allergic, inflammatory, injury-related, genetic, aging-related, and other (methods). The majority of the samples were from autoimmune diseases (40.8%), followed by healthy individuals (22.3%) and allergic disorders (13.1%) (Figure 2A).

Table S1. Bulk transcriptomic datasets included in this study, related to Figure 2 and STAR Methods

To assess the efficacy and accuracy of DeepAdvancer, we performed a systematic benchmarking analysis. DeepAdvancer was systematically compared against two widely used batch correction methods. Uniform manifold approximation and projection (UMAP) was used to visualize the reconstructed expression profiles. The results showed that sample clustering in the original data was primarily driven by batch effects (Figure 2B). Compared to limma and ComBat, DeepAdvancer improved the clustering of samples by biological category (Figures 2C–2E), supporting its effectiveness in removing technical variation. These observations were further supported by quantitative assessments of integration quality (Figures 2F and 2G).

To further assess the biological validity of the corrected expression data, we evaluated the accuracy of inter-class expression ratios after batch correction. Consistent with our previous results, the ratios derived from DeepAdvancer-corrected data exhibited the highest concordance with reference ratios (Figures 2H and S1A). In addition, when comparing the top 100 differentially expressed genes between each pair of classes, DeepAdvancer achieved the highest average overlap (mean = 71.1) with reference gene sets, outperforming limma (mean = 29.7), ComBat (mean = 26.0), and uncorrected data (mean = 24.5) (Figures 2I and S1B). In addition, validation using representative disease marker genes showed that established markers for systemic lupus erythematosus (SLE) (*IFI27* and *ISG15*) and atopic dermatitis (AD) (*CCL17* and *CCL22*) remained significantly upregulated in disease samples after correction, with expression patterns consistent with the reference data (Figures S2A and S2B), supporting the preservation of key biological signals. Furthermore, we conducted a more comprehensive comparison using a smaller psoriasis-focused dataset. Compared to multiple correction methods, including limma, ComBat, Rank-In, RUVSeq, DeepComBat, and MNN,^13^^,^^14^^,^^15^^,^^16^ DeepAdvancer showed better overall performance (Figures S2C–S2E). Considering the imbalance in disease composition, we further performed a downsampling analysis by reducing the proportion of autoimmune disease samples from 40.8% to 20%, and then repeated the analysis under this more balanced setting (Figure S2F). The results showed that the inferred gene expression trends remained highly consistent with those obtained from the original dataset. Overall, these results indicate DeepAdvancer can effectively integrate large-scale and multi-platform omics datasets while reducing technical variation and preserving biological signals. These results highlight the potential of DeepAdvancer in pan-disease and pan-tissue omics analyses.

To evaluate computational scalability, we performed benchmarking analyses across datasets of varying sizes under a fixed training configuration (batch size = 128, epochs = 100, genes = 15,000). Training time increased approximately linearly with dataset size, while peak GPU memory usage ranged from 1.43 GB (1,000 samples) to 6.463 GB (10,000 samples) (Table S2). These results indicate that DeepAdvancer scales efficiently to large datasets. In practice, a GPU with ≥8 GB VRAM is sufficient for datasets up to ∼10,000 samples, demonstrating its practical feasibility for large-scale dataset applications.

### Innate immune programs are broadly activated across skin diseases

**Figure 3:**
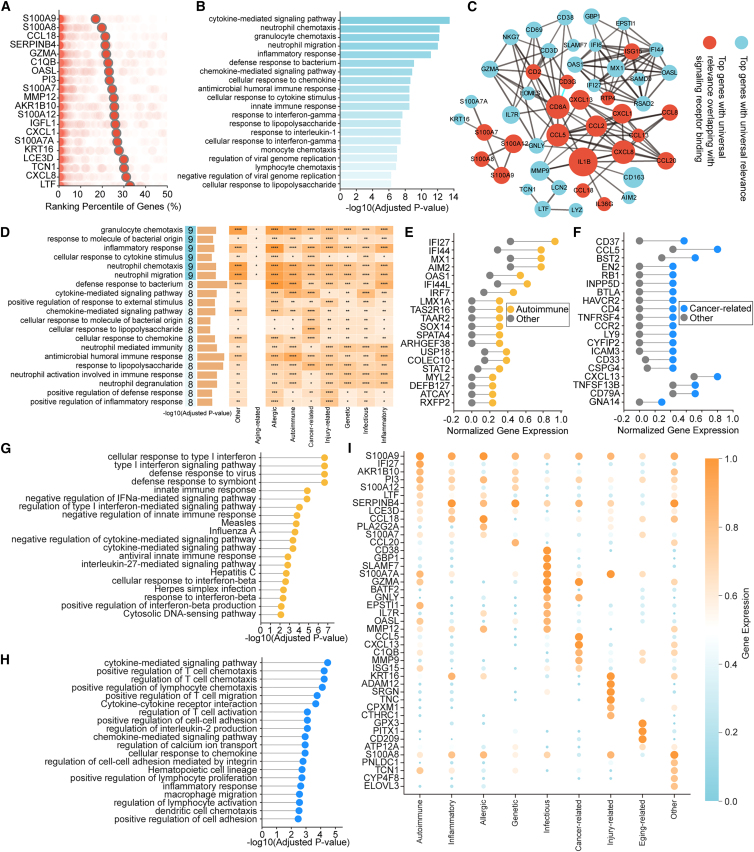
Universally and class-specific important genes and pathways across skin diseases (A) List of the highest-scoring genes across all skin diseases. Percentile scores are shown for each class, with average scores highlighted. (B) The top enriched pathways for the top 100 universally important genes. (C) Network of top 100 universal important genes. The network was generated with StringDB and disconnected genes are excluded. The size of each node was determined by the number of edges. Genes that are included in signaling receptor-related pathways are colored red. (D) List of top pathways that are universally important. The heatmap on the right shows the enrichment level of these pathways across different disease types. Color indicates the proportion of significant subclasses in each disease category enriched for the indicated pathway, with darker colors representing higher proportions. Asterisks indicate the significance of pathway enrichment, assessed by Fisher’s exact test with Benjamini–Hochberg correction for multiple comparisons: ∗*p* < 0.05, ∗∗*p* < 0.01, ∗∗∗*p* < 0.001, and ∗∗∗∗*p* < 0.0001; ns, not significant. (E and F) The plots of class-specific genes shown for autoimmune class (E) and cancer-related class (F). The colored dots show the percentile score of one gene for the specific disease type and the gray dots show the highest percentile score the same gene has among all the other types. The genes are sorted on the basis of the difference values. (G and H) The top enriched pathways for the class-specific genes for autoimmune class (G) and cancer-related class (H). (I) The marker genes for each class.

Using the unified expression data generated by DeepAdvancer, we first identified genes that consistently ranked highly across all skin diseases. These genes were primarily involved in skin inflammation and barrier damage (Figures 3A and 3B), such as *S100A8*, *S100A9*, *KRT16,* and *LCE3D*. Notably, *S100A8* and *S100A9* contribute to barrier-associated immune responses by promoting neutrophil recruitment and are widely recognized as damage-associated molecular patterns in inflammatory skin diseases such as eczema and contact dermatitis, where they amplify innate immune activation and drive disease progression.^17^ In autoimmune skin diseases such as psoriasis, they further stimulate pro-inflammatory pathways, including IL-17 and TNF-α signaling.^18^ Moreover, these genes are involved in host defense during cutaneous infections, and in skin cancers such as melanoma,^19^ they have also been proposed as potential diagnostic or prognostic markers.

To better understand how these genes interact at the systems level, we performed network analysis. This revealed a core module dominated by innate immune signaling, enriched for hub genes involved in cytokine-receptor interactions (Figure 3C). Pathway enrichment analysis further highlighted innate immune processes that were recurrent across diseases, including granulocyte chemotaxis, response to bacterial molecules, and inflammatory response (Figure 3D). This widespread activation shows that skin, as a frontline barrier organ,^20^ relies on rapid and nonspecific immune responses, underlining a common immune activation program across skin diseases.

Next, we aimed to identify type-specific genes (Figures 3E–3G). We found that the top autoimmune-specific transcripts were mainly interferon-stimulated genes (such as *IFI27*, *MX1*, *OAS1*) (Figures 3E and 3G),^21^ suggesting that interferon-mediated chronic immune activation may represent an important mechanism underlying autoimmune diseases.^22^ By contrast, in skin malignancies, the selectively elevated genes were involved in T cell activation, co-stimulatory signaling, and antigen presentation (Figures 3F and 3H), underscoring the central role of adaptive immunity within the tumor microenvironment.^23^ In infectious skin diseases, DeepAdvancer identified the elevated expression of *IRF8*, *GBP1*, *GBP4*, *CD53*, and *IL18RAP* (Figure S3A), indicating that innate immune and antimicrobial responses were activated in these diseases.^24^ Top allergic-specific genes (Figure S3B) comprised transcripts associated with chemokines, alongside barrier repair genes, depicting a type 2 immunity-dominated hypersensitivity response.^25^ Moreover, genes (Figure S3C) upregulated in inflammatory diseases were involved in MAPK signaling and neuroimmune regulation, suggesting a chronic low-grade inflammatory state. In injury-related conditions, strong induction of extracellular matrix (ECM) remodeling genes (Figure S3D) indicated active tissue repair and fibrotic processes.^26^ Genetic skin diseases displayed a more complex transcriptional landscape, with class-specific genes (Figure S3E) linked to epidermal structure maintenance, neural signaling, and developmental regulation, suggesting the dysregulation of the “structural-neuronal-developmental” axis.^27^ In aging-related skin conditions, upregulated genes (Figure S3F) were enriched in oxidative stress responses, mitochondrial metabolism, and cell cycle regulation, reflecting the impact of ROS accumulation and impaired antioxidant defenses.^28^ These findings were further supported by marker gene analysis across different types (Figures 3I and S3G).

In summary, we identified a common skin inflammation program dominated by innate immune activation and uncovered type-specific immune genes that may inform diagnosis and guide targeted interventions. These results also validate the effectiveness of our approach, demonstrating that DeepAdvancer captures key biological processes.

### A transcriptomic continuum of skin diseases

**Figure 4:**
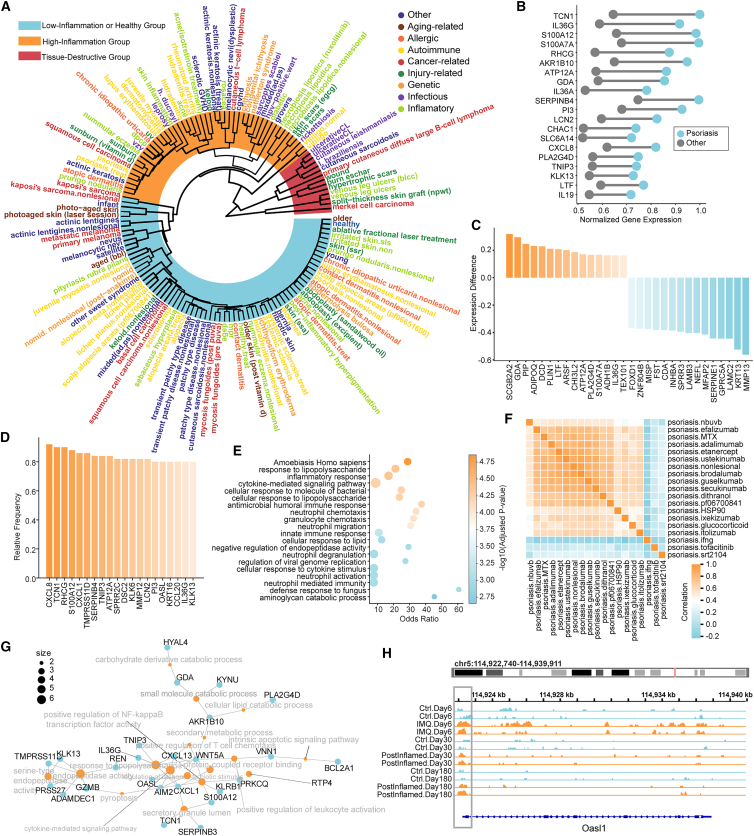
Integrated molecular profiling of skin diseases (A) Clustering analysis of various skin diseases based on transcriptomic signatures. (B) Abnormally upregulated genes associated with psoriasis. (C) Differentially expressed genes between psoriasis and cutaneous squamous cell carcinoma (SCC). (D) Genes consistently downregulated across different psoriasis treatments. (E) Functional enrichment analysis of the consistently downregulated genes. (F) Similarity matrix between different therapeutic modalities based on gene expression profiles. (G) Shared differentially expressed genes between various treatments and the non-lesional skin baseline. (H) Chromatin accessibility landscape around the *Oasl1* gene locus across different time points.

To systematically investigate the relationships among various skin diseases, we conducted a comparative analysis across all categories (Figure 4A). We observed that subtypes of the same disease often clustered together, such as primary melanoma and metastatic melanoma, suggesting that DeepAdvancer can capture disease-specific gene expression patterns. Notably, although skin diseases were classified as autoimmune, cancer-related, or infectious, the clustering patterns did not strictly follow these predefined categories. A typical example was that psoriasis was closer to actinic keratosis, AD, and squamous cell carcinoma (SCC) than to other autoimmune diseases.^29^ We found that transcriptional changes across skin diseases occurred along a continuum. This continuum could be broadly divided into three states: (1) a healthy or low-inflammation group characterized by immune regulation and tissue homeostasis (Figure S4A), including normal skin, non-lesional areas, healing-stage samples, and some low-inflammation tumor samples; (2) a high-inflammation group characterized by widespread immune activation (Figures S4B and S4C), including diseases such as psoriasis, lupus, rheumatoid arthritis, and eczema; and (3) a tissue destruction group characterized by co-activation of innate and adaptive immune responses, tissue remodeling, and structural breakdown, including necrosis, trauma, graft-related responses, and aggressive cancers (Figure S4D).

Interestingly, psoriasis and SCC exhibited similar patterns of transcriptional dysregulation.^30^ In psoriasis, we observed the upregulation of genes associated with inflammatory responses and immune regulation (Figure 4B). Similarly, SCC showed the elevated expression of pro-inflammatory signaling and epithelial differentiation genes (Figure S4E). These molecular signatures are consistent with their shared clinical manifestations, such as epidermal hyperplasia, abnormal keratinocyte differentiation, and chronic inflammatory infiltration. However, these shared features failed to conceal the fundamental differences in disease trajectories. We found that, unlike SCC, psoriasis specifically upregulated a set of genes involved in maintaining skin barrier integrity and lipid metabolism,^31^ such as *SCGB2A2*, *ADIPOQ*, *PLA2G4D*, *ATP12A*, and *CHI3L2*. In contrast, SCC upregulated genes were related to ECM degradation, tumor invasion, and proliferative signaling (Figure 4C),^32^ such as *MMP13*, *KRT13*, *SERPINE1*, *LAMC2*, and *INHBA*. These differences suggest a potential divergence in how keratinocytes respond to and regulate inflammatory stress in the local microenvironment. In psoriasis, keratinocytes remain capable of maintaining barrier function and metabolic homeostasis despite persistent inflammatory stimuli.^33^ By contrast, in SCC, inflammatory signaling may drive keratinocytes toward structural instability and invasive reprogramming.^34^ Thus, although both conditions are driven by inflammatory pathways, psoriasis represents a non-malignant inflammatory state with preserved barrier-associated programs, whereas SCC follows a trajectory of inflammation-induced remodeling, leading to barrier dysfunction and malignant transformation. This inflammation-dysregulation-transformation axis may underlie the fundamental divergence in disease fate between psoriasis and SCC.

In addition, psoriasis is a prototypical relapsing disease.^35^ Patients often experience recurrence after the cessation of treatment. To investigate the molecular basis of psoriasis relapse, we analyzed gene expression changes in lesional skin with different therapeutic interventions. We found that although treatment reduced the expression of many inflammation-related genes (Figures 4D–4F), some genes remained elevated compared to unaffected areas (Figure 4G).^36^ These genes are closely associated with inflammatory response, lipid metabolism, and T cell recruitment. Among them, *AIM2* has previously been implicated in the establishment of immune memory following skin damage.^37^ In the original study, ATAC-seq was performed on normal mouse skin, imiquimod-induced inflamed skin, and skin collected at multiple time points after inflammation resolution (day 6, day 30, and day 180). By analyzing the original ATAC-seq dataset, we reproduced the previously reported result that the promoter region of the *Aim2* locus remained highly accessible even 180 days after inflammation had resolved (Figure S4F). Intriguingly, we also observed that chromatin accessibility at the *Oasl1* gene locus remained elevated at day 180 after the resolution of inflammation (Figure 4H),^38^ suggesting persistence of an epigenetically primed state at selected inflammatory loci. Targeting these persistent epigenetic programs may be essential for breaking the cycle of psoriasis relapse. Together, our analysis delineates a transcriptomic continuum of skin diseases, revealing shared immune circuits and molecular determinants that shape divergent clinical trajectories, thereby providing a foundation for future mechanistic and translational studies.

### stNiche reveals niche dynamics across developmental, homeostatic, and diseased human skin

**Figure 5:**
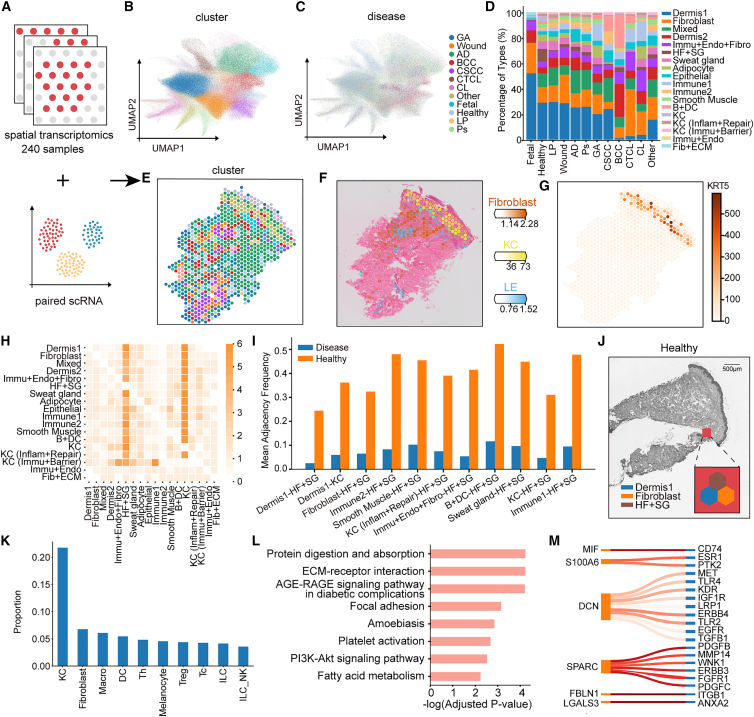
Identification and characterization of spatial niches in human skin using stNiche (A) Schematic overview of the integration of ST and matched single-cell RNA-seq datasets. (B) UMAP visualization of distinct cellular clusters. (C) Distribution of cellular subpopulations across various skin conditions. (D) Stacked bar plot showing changes in cellular composition across different diseases. (E–G) Cell-type deconvolution of ST spots (E) using Cell2Location based on scRNA-seq annotations, with spatial mapping of inferred cell types (F) and corresponding marker gene expression (G). (H) Heatmap showing differences in second-order structures between healthy and diseased samples. (I) Bar plot showing significantly altered second-order spatial interactions. (J) Spatial niche identified by stNiche, composed of three co-localized spot types enriched for hair follicle, fibroblasts, and immune cells. Scale bars, 500 μm. The magnified inset is for illustrative purposes only and is not shown to scale. (K) Proportions of major cell types within the identified niche. (L) Enriched functional pathways associated with the niche. (M) Ligand–receptor interaction network highlighting potential signaling between the niche and surrounding microenvironment.

To further investigate how gene expression programs are spatially organized in the skin, we analyzed ST profiles from 240 samples (Figure 5A and Table S3). UMAP projection revealed 17 distinct cellular subpopulations (Figure 5B) across various pathological states (Figure 5C). We found fetal samples were enriched in dermal and fibroblast populations, consistent with an active developmental state. In contrast, healthy adult skin exhibited a more stable architecture. In addition, wound samples showed elevated fibroblast content and increased immune cell infiltration, suggesting the activation of tissue repair and inflammatory programs. In psoriasis and AD, epidermal and immune cell populations expanded significantly, indicative of widespread immune activation and epidermal dysregulation. Tumor samples showed remodeling of the local microenvironment, with marked shifts in cellular composition (Figure 5D).

To achieve spatially resolved cell-type localization, we applied Cell2location^39^ to decompose cell-type composition across ST spots via annotated scRNA-seq references. The results revealed clear spatial localization patterns for key cell types (Figure 5E), including fibroblasts, keratinocytes, and luminal epithelial cells (Figure 5F). The predicted distributions closely mirrored the spatial expression patterns of marker genes such as *KRT5* (Figure 5G), supporting the accuracy of the inferred cellular composition.

Next, we investigated disease-associated alterations in spatial tissue organization using stNiche (Methods), and found that several second-order structural connections were significantly reduced in disease samples compared to healthy controls (Figures 5H and 5I). Notably, stNiche identified a niche comprising three spatially co-localized spot types enriched for hair follicle, fibroblasts, and immune cells (Figures 5J and 5K). This observation is consistent with previous histological evidence supporting the existence of a functional hair follicle-associated niche in healthy skin. By contrast, conventional spatial domain identification methods mainly captured within-section spatial domains and were less effective in resolving the cross-condition niche identified by stNiche (Figure S5A).^9^^,^^40^ Functional enrichment analysis revealed significant enrichment in ECM-receptor interaction, AGE-RAGE signaling, focal adhesion, and the PI3K-Akt pathway (Figure 5L).^41^^,^^42^^,^^43^ These pathways are known to mediate processes such as cell migration, proliferation, ECM remodeling, and inflammatory response, suggesting that this niche may serve as a hub for tissue repair and immune modulation. Furthermore, ligand-receptor interaction analysis of stNiche uncovered a signaling network centered around *MIF*, *S100A6*, *DCN*, and *SPARC*, which interact with cell-surface receptors such as *CD74*, *ESR1*, and *TGFBR1* (Figure 5M). These interactions may contribute to epithelial repair, matrix reorganization, and local inflammatory activation. Together, these results suggest that loss of this niche may be associated with inflammation-related structural deterioration in skin diseases.

We further applied stNiche to psoriasis samples and identified a niche composed of dermis1 and immune2 cell clusters (Figure S5B). Functional enrichment analysis revealed that this niche was enriched in lipid metabolism and inflammatory pathways (Figure S5C),^44^ consistent with our prior findings (Figure 4G). In basal cell carcinoma (BCC), we identified a niche consisting of dermis2 and “Immu+Endo+Fibro” cell clusters (Figure S5D), enriched for cancer-associated and endocrine/metabolic pathways, suggesting a multifunctional role in immune evasion (Figure S5E). In fetal skin, a niche formed by dermis1 and fibroblasts (Figure S5F) was enriched for pathways related to ECM remodeling, PI3K-Akt signaling, and cell adhesion and migration (Figure S5G),^45^ pointing to its involvement in structural development and local tissue organization. Together, these findings indicate that spatial niches exhibit high state-specificity across developmental, homeostatic, and pathological conditions, and that niche remodeling may contribute to disease progression. Moreover, they highlight the utility of stNiche for integrative comparisons across multiple diseases and tissue sections.

### BioinAI provides a comprehensive analysis platform for bulk transcriptomic data

Based on large-scale integrative analyses across different scales, we explored the relationships among skin diseases along a continuous spectrum and identified key spatial structures. In contrast to previous integrative studies, our work not only developed analytical approaches but also implemented improvements in data preprocessing and analysis workflows.

**Figure 6:**
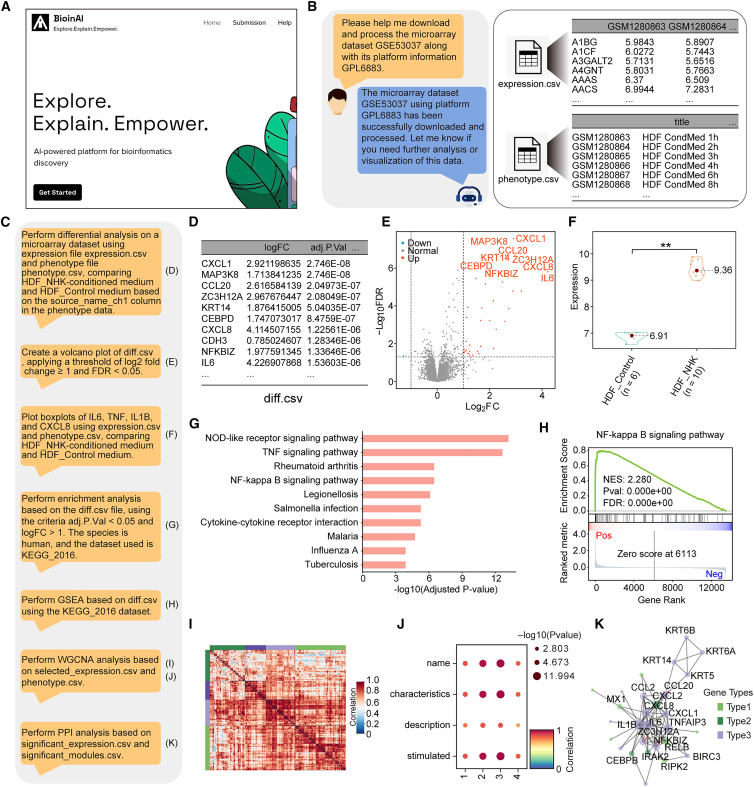
BioinAI-Web enables automated analysis of bulk transcriptomic data (A) Screenshot of the BioinAI online platform. (B) Example conversation illustrating a user request to download and preprocess transcriptomic data (left), with the resulting standardized gene expression matrix and phenotype metadata (right). (C) Sequence of user prompts and corresponding results. (D) Output of differentially expressed genes. Differential expression analysis was performed using limma based on a moderated *t* test, and *p* values were adjusted for multiple testing using the Benjamini–Hochberg method. (E) Volcano plot showing the distribution of significantly upregulated and downregulated genes. (F) Violin plots comparing expression levels of representative inflammation-related genes across two groups. Each dot represents one sample. Statistical significance was assessed by Student’s *t* test. ∗∗*p* < 0.01. (G) Bar plot of enriched functional terms among upregulated genes. Functional enrichment analysis was performed using the Fisher’s exact test, and *p* values were adjusted for multiple testing using the Benjamini–Hochberg method. (H) Gene set enrichment analysis highlights significant upregulation of the NF-κB pathway. Statistical significance was assessed using permutation-based gene set enrichment analysis. Normalized enrichment score (NES), nominal *p* value, and false discovery rate (FDR) are shown. (I) Heatmap displaying weighted gene co-expression network analysis (WGCNA) module assignment for differentially expressed genes. (J) Correlation between gene modules and clinical traits. Correlation coefficients were calculated using Pearson’s correlation analysis; dot size indicates −log10(*p* value), and color indicates the correlation coefficient. (K) Protein–protein interaction network of top differentially expressed genes.

We developed the BioinAI online platform (hereafter referred to as BioinAI-Web), which enables automated omics analysis through a conversational interface (Figure 6A), thereby reducing analysis time and manual effort. Users can initiate analysis workflows, upload datasets, and download results through natural language commands (Figure S6A). Specifically, BioinAI-Web supports automated dataset retrieval and preprocessing. Based on the experimental design and data type, the system automatically selects predefined normalization and gene-symbol harmonization procedures (Figure S6B), generating standardized gene expression matrices and corresponding phenotype tables (Figure 6B). During analysis, BioinAI-Web accommodates a range of common omics tasks (Figure 6C). For instance, the platform automatically performs differential expression analysis, with volcano plots illustrating the distribution of differentially expressed genes (Figures 6D and 6E). In addition, boxplots allow intuitive comparisons of key gene expression between groups (Figure 6F). The platform also integrates a suite of downstream analytical modules, including functional enrichment analysis (Figure 6G), gene set enrichment analysis (Figure 6H), weighted gene co-expression network analysis (Figures 6I and 6J), and PPI network reconstruction (Figure 6K).

To evaluate the utility and performance of BioinAI-Web, we conducted two exemplary analyses using the microarray dataset GEO: GSE53037 and the RNA-seq dataset GEO: GSE114729.^46^^,^^47^ In GSE53037, differential expression analysis revealed significant upregulation of genes associated with inflammation and cell migration in fibroblasts co-cultured with keratinocytes (Figures 6E and 6F),^48^ indicating that keratinocyte-fibroblast interactions can activate pro-inflammatory and migratory programs. Functional enrichment analysis of these genes highlighted inflammatory pathways such as NOD-like receptor, TNF, and NF-κB signaling (Figures 6G and 6H). Co-expression network analysis identified four gene modules (Figure 6I), among which module 3 showed the strongest association with the co-culture condition (Figure 6J) and included key genes such as *COPS3*, *SIRPA*, and *TNFAIP2* (Figure S6C). PPI analysis further revealed that these inflammatory mediators formed a densely interconnected signaling network (Figure 6K), underscoring their coordinated role in shaping inflammatory responses. These findings suggest that fibroblasts, activated by keratinocyte-derived signals, may act as amplifiers of cutaneous inflammation.

In GSE114729, BioinAI-Web generated standardized expression matrices and phenotype annotations across pre-treatment, post-treatment, and relapse stages of psoriasis. Comparative analysis revealed significant upregulation of lipid metabolism genes in relapse (Figures 4G, S6D, and S6E).^49^ In addition, pathway enrichment showed treatment suppressed pro-inflammatory pathways, including NOD-like receptor and cytokine receptor interactions, while enhancing drug metabolism pathways (Figures S6F–S6I). However, these inflammatory pathways became reactivated after drug withdrawal (Figures S6H and S6J). These findings once again suggest that the persistent dysregulation of lipid metabolism and reactivation of inflammatory signaling may contribute to psoriasis relapse. Overall, these results highlight the utility of BioinAI-Web in integrating and extracting biologically meaningful insights from both microarray and RNA-seq datasets.

### BioinAI provides a comprehensive analysis platform for scRNA-seq and ST data

**Figure 7:**
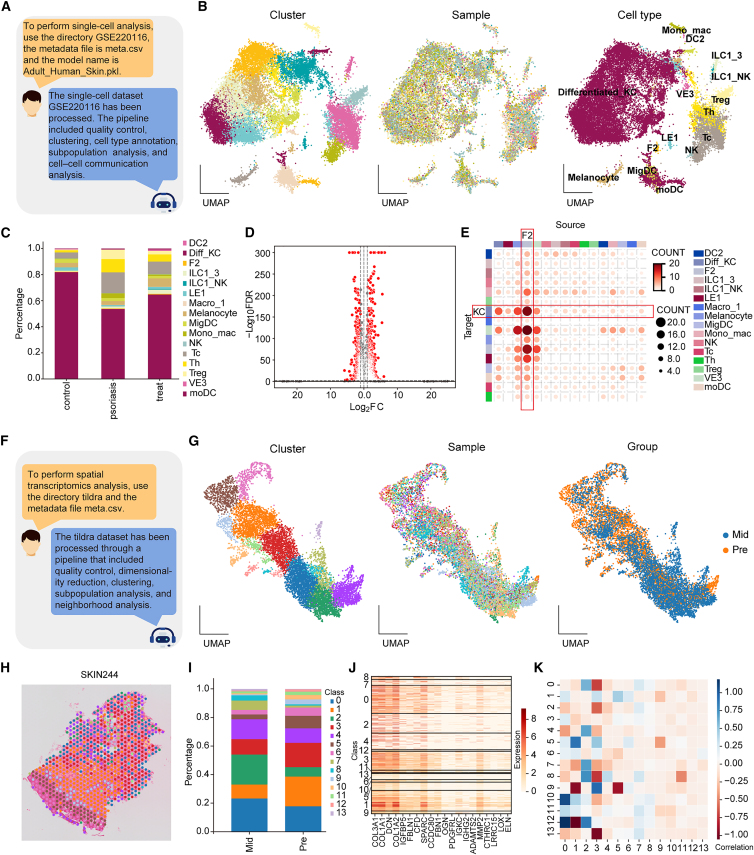
BioinAI-Web enables analysis of single-cell and spatial transcriptomics data (A) Representative query to the online platform requesting single-cell RNA-seq data processing and the corresponding response. (B) UMAP plots showing integrated single-cell data colored by cluster (left), sample (middle), and annotated cell types (right). (C) Stacked bar plots illustrating the proportion of cellular subpopulations across different groups. (D) Volcano plot showing differentially expressed genes in the keratinocyte subpopulation between healthy individuals and psoriasis patients. (E) Heatmap depicting intercellular communication changes among subpopulations in psoriasis. (F) Representative query to the online platform requesting spatial transcriptomics data processing and the corresponding response. (G) UMAP plots of integrated spatial transcriptomics data colored by cluster (left), sample (middle), and group (right). (H) Spatial mapping of subpopulations in psoriasis skin (sample Skin244). (I) Stacked bar plot showing changes in subpopulation composition before and after treatment. (J) Heatmap of marker gene expression in subpopulation 0. (K) Heatmap illustrating adjacency changes between subpopulations pre- and post-treatment.

BioinAI-Web also supports automated analysis of single-cell and spatial omics datasets (Figures 7 and S7). Users can initiate the analyses simply by submitting natural language commands (e.g., “analyze scRNA dataset GEO: GSE220116” or “process spatial transcriptomics dataset Tildra”).^7^^,^^50^ The system will then automatically launch the corresponding workflows. For single-cell datasets, BioinAI-Web executes a modular pipeline that includes quality control, batch correction, dimensionality reduction, clustering, cell type annotation, subpopulation proportion analysis, differential expression analysis, and cell-cell communication inference. If users wish to define custom experimental groups, they can upload grouping files along with the data, and the platform will automatically adapt to perform stratified analyses.

As a practical example, we analyzed dataset GSE220116 (Figure 7A), which includes single-cell profiles from psoriatic skin before and after treatment, along with healthy controls. BioinAI-Web integrated the data and constructed a UMAP embedding colored by cluster, sample origin, and annotated cell types (Figures 7B and S7A). Multiple subpopulations were identified, including keratinocytes, fibroblasts, and immune cells (Figures 7C and S7B). Differential expression analysis was performed across all subtypes by BioinAI-Web (Figure 7D). Cell-cell communication analysis revealed markedly enhanced interactions between “KC” and “F2” clusters in psoriatic skin (Figures 7E and S7C),^51^ which were notably reduced after treatment (Figure S7D). These interactions were centered around pro-inflammatory ligand-receptor pairs, including CXCL14_CXCR4 and CD44_TYROBP (Figure S7E). Consistent with our previous findings, these results further support that fibroblast-keratinocyte interactions play a pivotal role in amplifying local inflammatory signaling and driving epidermal dysregulation in psoriasis.

For spatial omics analysis, we evaluated the platform using the Tildra dataset (Figure 7F). BioinAI-Web automatically performed quality control, dimensionality reduction, clustering, and neighborhood graph analysis (Figure 7G). It visualized the spatial distribution of clusters in a psoriasis patient (Figure 7H) and quantified changes in spatial subpopulation composition before and after treatment (Figure 7I). Further marker gene and spatial adjacency analyses (Figures 7J and 7K) revealed spatial remodeling of fibroblast-associated clusters during treatment. Together, these results show that BioinAI-Web can be applied to bulk, single-cell, and spatial transcriptomic data.

## Discussion

In this study, we developed BioinAI, an integrated framework for multi-scale omics data analysis, comprising an online platform and two methods: DeepAdvancer and stNiche. Our results indicate that these two methods are effective for cross-platform data integration, biological signal extraction, and spatial niche characterization. In addition, the BioinAI online platform provides a user-friendly and fully automated analysis ecosystem, streamlining the entire workflow from data retrieval and preprocessing to downstream interpretation.

In large-scale omics data integration, effectively removing batch effects while retaining meaningful biological signals remains a major challenge, particularly across heterogeneous platforms. To address this, we developed DeepAdvancer, a class-aware adversarial autoencoder model designed for cross-platform data harmonization. DeepAdvancer establishes a unified embedding space for omics data across diverse platforms. Compared with traditional methods, it captures disease-relevant features more effectively under high heterogeneity. To benchmark its performance, we assembled a human skin transcriptome compendium comprising 49,738 samples across 1,016 datasets, which we systematically annotated into 132 subclasses and 10 major disease categories. Applying DeepAdvancer to this compendium, we uncovered a transcriptomic continuum of skin diseases characterized by a gradient of inflammatory activation,^52^ which provides a framework to position diseases along a shared spectrum rather than as isolated entities. Notably, psoriasis and SCC shared similar dysregulated immune signatures, including chemokine activation and epithelial remodeling.^29^^,^^30^ However, psoriasis retained barrier-related gene expression, suggesting an “adaptive inflammation” state—a non-destructive adaptation to sustained immune activation pressure.^33^ In contrast, SCC showed the upregulation of invasion-associated genes, indicating a shift toward a “remodeling-invasion” axis.^32^ This comparison illustrates how the continuum framework can resolve disease-specific trajectories and highlights its utility in explaining divergent clinical outcomes. Overall, our method enables a unified transcriptomic analysis of the entire dataset, offering insights from a more comprehensive perspective.

Psoriasis is a prototypical relapsing inflammatory disease, and our findings suggest that dysregulated lipid metabolism may play a key role in its relapse. Clinical studies have established strong associations between aberrant cholesterol and fatty acid metabolic pathways and psoriasis pathogenesis.^53^ Spatial transcriptomics further revealed upregulated lipid metabolism genes in sebaceous gland regions of psoriatic skin.^54^ Although direct experimental evidence linking lipid metabolism to relapse remains limited, recent commentary emphasizes that dysregulated lipid metabolism may be a critical non-genetic contributor to psoriasis recurrence.^49^ Moreover, the concept of metabolic reprogramming, reinforcing inflammatory memory, provides a plausible mechanistic framework for how sustained lipid metabolism abnormalities may fuel disease relapse.

For integrative analysis of single-cell and spatial omics data across slides, we developed stNiche, which applies a spatial graph learning framework to identify functionally coordinated cellular niches across tissue states. In healthy skin, we identified a follicle-associated niche composed of immune cells, fibroblasts, and epithelial components.^55^ This niche is enriched for signaling pathways related to stromal remodeling, epithelial repair, and immunomodulation. We propose that it may function as a spatially organized unit that preserves tissue integrity through immune regulation within the local microenvironment. In disease contexts such as psoriasis and BCC, we observed distinct architectures with altered functions.^56^ These findings suggest that disease progression involves not only cell-type changes but the disintegration and reprogramming of spatially defined microenvironments, highlighting niche remodeling as a key hallmark of pathogenesis.

Our large-scale data mining efforts were enabled by the BioinAI-Web, which integrates a semi-agent system with LLMs. This design supports natural-language interaction, modular workflow execution, and stable outputs, while mitigating common issues such as LLM hallucinations.^11^ Compared with fully model-driven systems,^57^ BioinAI-Web offers a complementary approach that balances automation with interpretability and analytical control. Benchmark analyses indicate the platform’s scalability and versatility in integrating high-throughput datasets and extracting biological insights. Future work will focus on enhancing multi-omics integration, improving agent-user interactions, incorporating more advanced analytical algorithms, and strengthening experimental validation. Together, BioinAI-Web represents a step toward the automation, standardization, and intelligent transformation of omics research, providing a useful framework for dissecting disease mechanisms and enabling next-generation scientific exploration.

### Limitations of the study

Despite these advances, the current framework still has several limitations. First, although DeepAdvancer performed well across large-scale public datasets, its evaluation was primarily based on computational benchmarking and retrospective integration. Thus, further prospective and experimental validation is required. Second, the reconstructed expression landscape may be influenced by sample composition, annotation quality, and uneven representation of disease subtypes. Third, stNiche may be influenced by spatial resolution, annotation accuracy, and graph construction strategies. Finally, although BioinAI-Web improves automation and usability, its performance still depends on the quality of input data, embedded workflows, and reference resources.

## Resource availability

### Lead contact

Further information and requests for resources should be directed to and will be fulfilled by the lead contact, Prof. Kun Chen (chenk@tongji.edu.cn).

### Materials availability

This study did not generate new biological materials or proprietary reagents.

### Data and code availability

• Public bulk transcriptomic datasets related to human skin were retrieved from Gene Expression Omnibus (GEO). Public human skin spatial transcriptomic datasets were collected through systematic database searches. Input gene expression datasets, reconstructed expression profiles, and integrated spatial transcriptomics datasets are available on Figshare: https://doi.org/10.6084/m9.figshare.29589959.^58^ All dataset accession numbers are provided in the supplemental tables.
• The BioinAI framework developed in this study integrates DeepAdvancer, stNiche, and the BioinAI-Web online platform for transcriptomic data analysis. BioinAI-Web is accessible at https://www.bioinai.com. Original code is publicly available at Figshare: https://doi.org/10.6084/m9.figshare.32076603.^59^ The corresponding source repositories are available at https://github.com/BioinAI/DeepAdvancer and https://github.com/BioinAI/stNiche.
• Any additional information required to reanalyze the data reported in this study is available from the lead contact upon request.

## Acknowledgments

The work was financially supported by the National Natural Science Foundation of China (no. 32370961), the Shanghai Rising-Star Program (no. 21QA1407600), the Open Research Fund of Basic Medicine College (no. JCKFKT-ZD-004), and the 10.13039/501100012226Fundamental Research Funds for the Central Universities (nos. 22120250158 and 22120250374).

## Author contributions

K.C. designed and supervised the research. M.C. and S.W. organized and analyzed the data. M.C. developed the framework, created the figures, and drafted the manuscript. F.Y. and Y.W. proofread and revised the manuscript. M.C., F.K., N.K., M.L., X.Q., Z.X., Z.Y., Y.Y., and J.Z. participated in the development and testing of the online platform. All authors read, verified the data, and approved the final version of the manuscript. K.C. was responsible for the decision to submit the manuscript.

## Declaration of interests

The authors declare no competing interests.

## Declaration of generative AI and AI-assisted technologies in the writing process

During the preparation of this work, the authors used ChatGPT (OpenAI) to improve the readability of some text. The authors reviewed and edited the content and take full responsibility for the publication.

## STAR★Methods

### Key resources table

**Table:** 

REAGENT or RESOURCE	SOURCE	IDENTIFIER
**Deposited data**		

Public bulk transcriptomic datasets of human skin	GEO	GEO accession numbers are listed in Table S1
Public human skin spatial transcriptomic datasets	Public repositories/published studies	Dataset accession numbers are listed in Table S3
Curated spatial transcriptomic dataset collection	This study	Figshare: https://doi.org/10.6084/m9.figshare.29589959

**Software and algorithms**		

DeepAdvancer	This study	GitHub: https://github.com/BioinAI/DeepAdvancer; Figshare: https://doi.org/10.6084/m9.figshare.32076603
stNiche	This study	GitHub: https://github.com/BioinAI/stNiche; Figshare: https://doi.org/10.6084/m9.figshare.32076603
BioinAI-Web online platform	This study	https://www.bioinai.com/
AgentScope	agentscope-ai	version 1.0
Scanpy	scverse	version 1.10.1
Cell2location	BayraktarLab	version 0.1.5
Squidpy	scverse	version 1.8.1
limma	Bioconductor	version 3.66.0
removeBatchEffect	limma	function in limma 3.66.0
ComBat	sva	function in sva 3.58.0
Sva	Bioconductor	version 3.58.0
RUVSeq	Bioconductor	version 1.44.0
MNN/fastMNN	batchelor	version 1.26.0
DeepComBat	hufengling	https://doi.org/10.1002/hbm.26708
Rank-In	Tang et al. (2021)	https://doi.org/10.1093/nar/gkab554
STAGATE	zhanglabtools	https://doi.org/10.1038/s41467-022-29439-6
SpaGCN	jianhuupenn	version 1.2.7
R	R Foundation for Statistical Computing	version 4.5
Bioconductor	Bioconductor	release 3.22
Python	Python Software Foundation	version 3.11

### Method details

#### Bulk transcriptomic data preprocessing

To ensure cross-dataset comparability, all expression matrices were harmonized using unified gene symbols. Genes with missing values in more than 20% of datasets were removed. Datasets with gene coverage below 80% of the unified gene set were excluded to improve data quality. To reduce technical noise and enhance downstream analytical robustness, extreme expression values were clipped only when the maximum expression value in a dataset exceeded 10 times the dataset mean expression value. In such cases, values were truncated to the 1st and 99th percentiles of the expression distribution within that dataset. Log2 transformation was applied when either the maximum expression value exceeded 100, or the expression range (maximum minus minimum) exceeded 50 while the first quartile remained greater than 0. Redundant samples with identical expression profiles were removed. Missing values were imputed using within-gene mean imputation across samples after dataset harmonization.

#### DeepAdvancer pre-training data preparation

To systematically quantify gene expression differences between reference classes and other disease classes, we developed and applied the BioinAI_FC module—a tool optimized for efficient logFC estimation across heterogeneous bulk transcriptomic datasets. The module employs a hierarchical strategy based on the degree of class co-occurrence within datasets and divides pairwise class relationships into three types: directly shared, indirectly shared, and non-overlapping. For directly shared pairs, in which both classes appear within the same dataset, the corresponding samples are extracted to construct design and contrast matrices. Differential expression analysis is performed using the limma package. For indirectly shared pairs, in which the two classes do not co-occur in the same dataset but both share an intermediate third class, the median expression of the intermediate class is used to bridge the two datasets. Batch effects are removed using limma-based correction prior to logFC computation. For non-overlapping pairs, in which no shared classes exist, datasets containing the individual classes are merged. After preprocessing and transformation, limma is also used to estimate differential expression after batch correction. This strategy is considered less robust due to potential bias and is used only when no shared classes are available.

To improve robustness, comparisons from multiple datasets were filtered based on Pearson correlation of dataset-level expression or logFC profiles. For directly shared categories represented by more than three datasets, dataset-specific logFC profiles were retained when their correlation with at least one other dataset was greater than or equal to 0.3; otherwise, all datasets were retained. This step was used as a permissive filter to remove strongly discordant datasets while retaining biologically informative comparisons. For indirectly shared comparisons, dataset matching was performed using Pearson correlation of the mean expression profiles of the intermediate shared class across datasets. Because these comparisons rely on cross-dataset bridging and are potentially more sensitive to batch-related bias, a stricter similarity criterion was applied. The top three matched datasets were retained for each candidate dataset, and pairs with similarity greater than 0.6 were preferentially selected. If no pair met this threshold, the top 10 highest-similarity pairs were retained. The final result is a multi-class logFC matrix serving as input for downstream tasks.

Prior to model training, the expression profile of baseline classes was further optimized to improve cross-class feature consistency. Initial baseline values were computed as the mean expression of the base classes across datasets. An optimization algorithm was then applied to refine the baseline profile using information from directly shared classes. Specifically, one-hop shared classes that co-occurred with the base class in at least one dataset were first identified. Corresponding logFCs between the base class and these shared classes were extracted. Then, the initial expression matrix was optimized to maximize expression pattern similarity of the same one-hop class as calculated from different baseline classes. Two types of sharing relationships were considered: (1) one-hop classes shared by all baseline classes, and (2) pairwise sharing between subsets of baseline classes. To prevent overfitting and drift from the original center, an L2 regularization term was added to constrain deviations from the initial mean vector. Optimization was performed for 100 iterations using gradient-based updates, with a fixed learning rate of 0.001 and a regularization weight linearly increased from 0.0001 to 0.001. This procedure generated a refined center matrix for downstream modeling.

To retain full gene expression information and support downstream modeling, the raw gene expression matrix was used directly as model input, without applying dimensionality reduction or feature selection. To improve inter-sample comparability and mitigate biases arising from differences in numerical scale, MinMax normalization was applied to each dataset independently, scaling gene expression values to the range [0,1]. This design encourages the model to learn stable representations and reduces gradient instability.

To improve the balance of training data, all samples were grouped into 14 platform categories based on technological similarity. Platforms with minimal sample counts or structural redundancy were merged to ensure adequate representation across categories. To enhance the interpretability and generalizability of downstream disease-level analysis, disease samples were further annotated into ten major clinical groups based on etiological mechanisms or shared clinical features. These included: (1) healthy controls (normal skin from unaffected donors or adjacent non-lesional control tissues); (2) autoimmune diseases (e.g., systemic sclerosis, lupus erythematosus); (3) allergy-related disorders (e.g., atopic dermatitis, chronic urticaria); (4) infectious diseases (bacterial, viral, or fungal infections of the skin); (5) genetic disorders (congenital or hereditary skin diseases); (6) injury-related conditions (e.g., trauma, burns, postsurgical healing); (7) aging-related conditions (e.g., photodamage, aging); (8) cancers (primary cutaneous carcinomas, skin lymphomas); (9) inflammatory conditions not clearly attributable to immune or infectious origins but showing pronounced skin inflammation; (10) others, including samples with ambiguous or undefined diagnostic categories. This disease reclassification was used for visualization only and was not involved in model training.

#### DeepAdvancer framework

The DeepAdvancer framework is a gene expression decomposition model that enables the reconstruction of expression profiles. Due to the high dimensionality and heterogeneity of transcriptomic data, the model is implemented as an autoencoder architecture, consisting of a nonlinear encoder and a decoder-derived gene-level signal matrix. Let *X*∈*R*^*N*×*M*^ represent the input gene expression matrix, where *N* is the number of samples and *M* is the number of genes. The encoder network *f*_∅_:*X*→*Z* maps the input data *X* to a latent representation *Z*∈*R*^*N*×*D*^, where *D* denotes the number of latent basis components. The encoder consists of a multilayer perceptron with hidden dimensions 512, 256, 128, and 64, with CELU activations and dropout regularization. The latent representation is modulated by a class-specific proportion tensor *P*∈*R*^*D*×*C*×*M*^, where *C*is the number of biological classes, and *M* is the number of genes. This tensor is derived from precomputed differential expression statistics. Instead of using the decoder as a conventional neural network for reconstruction, we derive a gene-level signal matrix *W* by collapsing all linear decoder layers into a single transformation. The reconstructed expression profile $\hat{X}$ is then computed from the adjusted latent components and the signal matrix:

$$\hat{X}=h\left(Z,P,W\right)$$

where *h*(·) denotes the class-specific components adjustment and gene reconstruction procedure implemented in the function calculate_gene_expression. The model is trained with auxiliary batch and class discriminators operating on the latent representation. The corresponding batch and class prediction losses were incorporated into the overall objective during training. The reconstruction loss is defined as:

$$L_{\text{recon}}={\left\|\hat{X}-X\right\|}_{2}^{2}$$

The batch and class losses are computed directly from the outputs of their respective discriminators. In addition, to encourage consistency between the decoder-derived signal matrix and the expected signal matrix estimated during pre-training, a signal-matrix loss was included:

$$L_{\text{sig}}={\left\|W-W_{\text{expected}}\right\|}_{2}^{2}$$

A latent class-center regularization term was used to reduce within-class dispersion in latent space:

$$L_{\text{center}}=\sum_{c=1}^{C}\sum_{z_{i}\in C_{c}}{\left\|z_{i}-\mu_{c}\right\|}_{2}^{2}$$

where *μ*_*c*_ denotes the running latent center of class *c*. An intermediate components-consistency loss was introduced to encourage coherent gene expression across latent components. The loss is defined as the mean squared pairwise distance:

$$L_{\text{intermediate}}=\frac{1}{ND^{2}}\sum_{i=1}^{N}\sum_{f_{1}=1}^{D}\sum_{f_{2}=1}^{D}{\left\|F_{i,f_{1}}-F_{i,f_{2}}\right\|}_{2}^{2}$$

where *F*_*i*_∈*R*^*D*×*M*^ denotes the component-wise gene contribution matrix for sample *i*. The full training objective is therefore formulated as:

$$L_{total}=L_{recon}+L_{sig}+L_{batch}+L_{class}+L_{center}+L_{intermediate}$$

This architecture was designed to reduce batch-related effects while preserving class-related expression characteristics. Model parameters were optimized using the Adam optimizer with a learning rate of 0.0001, a batch size of 128, and 100 training epochs.

#### Batch effect correction using multiple methods

To benchmark DeepAdvancer against other correction strategies, we applied a range of established batch effect correction methods to the input expression matrices. These methods span linear models, nonlinear mappings, and deep learning–based frameworks, representing diverse theoretical approaches to batch effect mitigation. The following methods were used: (1) ComBat, an empirical Bayes–based linear model widely applied in both microarray and RNA-seq studies. We incorporated disease categories as covariates in the design matrix to preserve biological variability while removing technical effects. (2) removeBatchEffect from the limma package, a fast linear adjustment method that fits gene-wise linear models and removes batch-associated components. This served as the default correction strategy in our primary modeling pipeline. (3) RUVSeq, which infers and removes unwanted variation using negative control genes or a set of presumed invariant genes. This method is particularly suited to RNA-seq datasets lacking explicit batch annotations. (4) Rank-In, a normalization method based on expression rank preservation across sample groups. It is stable in cross-platform correction tasks. (5) MNN (mutual nearest neighbors), which performs nonlinear alignment of samples by identifying mutual neighbors in latent expression space. (6) DeepComBat, a hybrid method that combines the feature extraction capabilities of deep neural networks with the statistical correction strengths of ComBat. For all methods, we ensured a consistent gene space and harmonized sample annotations. All benchmark methods were applied using the default parameter settings provided in their original implementations or software packages. For ComBat, disease category was included as a covariate in the design matrix to preserve biological signal during correction. Correction outcomes were systematically evaluated under a unified framework.

#### Evaluation of batch correction performance

To systematically assess the effectiveness of different batch correction methods, we performed both qualitative and quantitative evaluations on the corrected expression matrices using clustering consistency scores and biological signal preservation metrics. To qualitatively evaluate integration performance, we visualized samples in a low-dimensional space using UMAP. Effective batch correction is indicated by reduced clustering according to dataset origin and improved grouping according to biological labels.

We next evaluated the compactness and separation of sample groups in the reduced-dimensional space using either disease labels or batch labels. For each class, we computed a class-specific centroid and calculated two types of distance-based metrics for each sample: (1) the average distance to other samples of the same class, and (2) the average distance to the ten nearest centroids of other classes. These values were then used to compute a silhouette score, defined as:

s(i)=b(i)−a(i)max⁡(a(i),b(i))

where *a*(*i*)is the average intra-class distance and *b*(*i*) is the average nearest inter-class distance. Differences in silhouette coefficients between methods were assessed using Student’s *t* test. To further quantify batch mixing after correction, we applied the k-nearest neighbor batch effect test (kBET), which evaluates whether local neighborhood composition deviates from the expected batch distribution. Lower rejection rates indicate more effective removal of batch effects and better mixing of samples across datasets.

To further assess biological consistency after correction, we evaluated both differential gene consistency and expression ratio consistency. The overlap of the top 100 differentially expressed genes before and after correction was computed to assess the fidelity of signal retention, where higher overlap indicates better preservation of key biological signals. In addition, we computed the similarity between predicted and reference logFCs for one-hop shared class pairs using correlation analysis. Expression ratio consistency was further assessed by calculating the Spearman correlation between predicted gene expression ratios and reference ratios across disease classes, where higher correlation indicates better preservation of relative gene expression patterns.

#### Downstream analysis of corrected expression data

To identify shared genes, we first extracted expression profiles from all healthy samples as a reference baseline. For each disease class, differential expression was computed as log fold change relative to healthy samples. Within each disease group, genes were ranked based on log fold change, and the top 100 genes were selected. To evaluate the consistency of expression patterns across disease classes, genes were ranked within each class, and high-frequency genes were identified based on their recurrence among the top-ranked genes. The top 20 shared high-frequency genes were visualized. Functional enrichment analysis was performed on the top 100 genes for each disease using GO and KEGG. The top 20 enriched pathways were visualized. To identify disease-specific genes, we defined a specificity score for each gene as the difference between its rank in the target class and its best rank across all other classes. The top 20 genes with the highest specificity scores were defined as disease-specific genes. Functional enrichment analysis was further performed on these disease-specific gene sets.

#### Preprocessing of scRNA-seq and spatial transcriptomic data

For ST data, batch effect correction was first applied to integrate samples. Preprocessing was performed using Scanpy, including normalization, dimensionality reduction, and clustering. The resulting embeddings were visualized using UMAP. For the paired scRNA-seq data, preprocessing was also conducted using Scanpy, including quality control to remove low-quality cells, normalization, and dimensionality reduction. Cells were clustered and visualized using UMAP. Cell type annotation was performed using established marker genes curated from the literature, resulting in a high-confidence cell-type reference dataset. Cell type decomposition of the ST data was conducted using Cell2location, a probabilistic framework that leverages the annotated scRNA-seq reference to estimate the cell-type composition of each spatial spot. All preprocessing steps were performed using consistent default parameter settings across datasets to ensure comparability.

#### Spatial niche identification using stNiche

We developed stNiche, a framework for identifying tissue microenvironments based on ST data. First, adjacency relationships between spot clusters were extracted to construct a sample-specific adjacency matrix. Adjacency was defined between each spot and its immediate spatial neighbors. For any pair of cell cluster types *L*_*x*_ and *L*_*y*_, we computed the frequency of their adjacency $A_{L_{x},L_{y}}^{\left(s_{i}\right)}$ within each sample *s*_*i*_, and normalized it to obtain an adjacency proportion:

$$P_{L_{x},L_{y}}^{\left(s_{i}\right)}=\frac{A_{L_{x},L_{y}}^{\left(s_{i}\right)}}{N_{L_{x}}^{\left(s_{i}\right)}}$$

where $N_{L_{x}}^{\left(s_{i}\right)}$ denotes the total number of spots assigned to cluster *L*_*x*_ in sample *s*_*i*_. To identify differential adjacency patterns, adjacency proportions for each cluster pair were compared between two biological groups using a two-sided Mann–Whitney U test, followed by Benjamini–Hochberg correction for multiple testing. Significant adjacency pairs were further filtered by requiring enrichment in a predefined focus group, using a fold-enrichment threshold of 4.0 and an adjusted *p* value threshold of 0.05.

Based on the filtered significant second-order adjacency pairs, we constructed higher-order structures by expanding each pair to include neighboring spots within the same sample. Third-order structures were first generated by adding one adjacent spot to each significant pair. For each structure, a geometric signature was computed from the pairwise distances among its constituent spots together with the corresponding cluster-label pairs. Structures were grouped together if they shared identical geometric distance patterns and matched unordered cluster-label combinations. For each structure group *G*_*i*_, we computed its coverage ratio:

$$C_{G_{i}}=\frac{M_{G_{i}}}{M_{total}}$$

where $M_{G_{i}}$ is the number of samples in which structure group *G*_*i*_ is observed, and *M*_*total*_ is the total number of samples in the focus group. To identify enriched structures, we calculated FC in group-specific frequencies:

$$FC_{G_{i}}=\frac{{\bar{M}}_{G_{i}}}{{\bar{M}}_{\text{Other}}}$$

where ${\bar{M}}_{G_{i}}$ is the average occurrence count of structure group *G*_*i*_ across samples in the focus group, and ${\bar{M}}_{\text{Other}}$ is the average occurrence count of all other structure groups.

To further refine higher-order niches, an iterative expansion procedure was applied. In each round, candidate structures were expanded by adding one neighboring spot, regrouped according to geometry, and re-evaluated for enrichment, coverage, and statistical significance. If an expanded structure failed to satisfy the predefined criteria (including a fold change threshold of 4.0, an adjusted *p* value threshold of 0.05, and a minimum coverage threshold of 0.6), the last valid structure was retained. This hierarchical and statistically constrained strategy enables robust identification of spatial niches that capture recurrent and biologically meaningful tissue microstructures.

#### Functional and signaling analysis of spatial niches

After identifying spatial niche structures, we further characterized their cellular composition and functional relevance. Based on prior high-confidence cell type annotations from scRNA-seq, we determined the cell type composition within each niche region by Cell2location. To investigate niche-specific functional activity, we performed differential gene expression analysis between niche regions and non-niche spots from the same tissue samples. Differential gene expression analysis was performed using the Wilcoxon rank-sum test, and genes with adjusted *p* value <0.05 and logFC >1 were considered significant. Functional enrichment analysis was then conducted on these genes to reveal the biological processes and pathways associated with each niche type. To further explore communication between niches and their surrounding microenvironment, we used the Squidpy framework to infer spatial signaling interactions.^60^ Specifically, we assessed ligand–receptor activity between niche spots and adjacent spatial domains, enabling the reconstruction of spatially resolved intercellular communication networks.

#### Construction of the BioinAI online platform

To enable automated processing and intelligent analysis of bulk transcriptomic data, we developed the BioinAI online platform (accessible at https://www.bioinai.com) based on the AgentScope framework.^61^ The platform integrates collaboration among multiple semi-agents with LLM interfaces to support natural language-driven omics analysis. At the core of the backend system is a ReActAgent architecture, which orchestrates key analytical tasks including data retrieval, preprocessing, differential expression analysis, and feature integration. The frontend adopts a modular design, offering functionality for data upload, results download, and user support. Users interact with the platform through natural language prompts. The system interprets these requests and automatically executes the corresponding analysis pipeline, including matrix construction, normalization, model inference, and result generation. BioinAI-Web currently supports complete pipelines for bulk transcriptomic, single-cell transcriptomic, and spatial transcriptomic data.

### Quantification and statistical analysis

Quantification, statistical analysis, and visualization were performed using Python, R and Microsoft Excel. Differences in silhouette coefficients between methods were assessed using Student’s *t* test. Group-level differences in adjacency proportions were assessed using the Mann-Whitney U test. *p* values were adjusted for multiple comparisons using the Benjamini-Hochberg method. Functional enrichment analysis was performed using Fisher’s exact test, and *p* values were adjusted for multiple testing using the Benjamini–Hochberg method. Expression ratio consistency was evaluated using Spearman correlation. Significant differences are indicated as follows: ∗*p* < 0.05, ∗∗*p* < 0.01, ∗∗∗*p* < 0.001, ∗∗∗∗*p* < 0.0001. Additional details are provided in the method details section and figure legends.
