# Supplementary material for: An intelligent framework for advancing large-scale omics data integration
Source: iScience. 2026 Apr 29;29(6):115928. doi: 10.1016/j.isci.2026.115928 (PMC13196096; doi:10.1016/j.isci.2026.115928)
Supplement: Document S1. Figures S1–S7, Tables S2 and S3 [file mmc1.pdf]

**iScience, Volume *29***

## Supplemental information

# An intelligent framework for advancing large-scale omics data integration

**Mintian Cui, Shixi Wang, Fan Yang, Yifei Wang, Fanyu Kong, Ni Kong, Mengying Li, Xiaoyue Qiao, Zhen Xu, Ziyu Yan, Yu Yan, Jiamo Zhang, and Kun Chen**

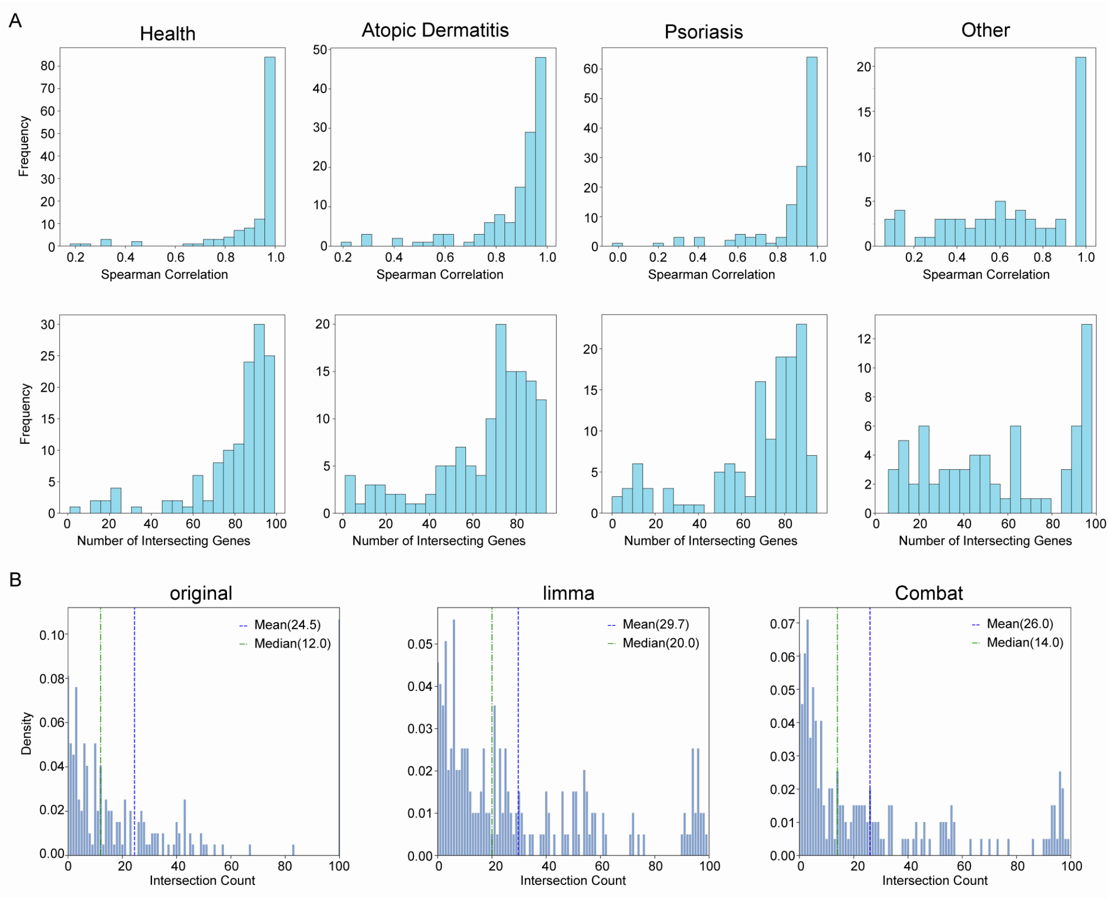


**Figure S1: Evaluation of expression ratio consistency and gene overlap. A**, Distribution of spearman correlation coefficients and gene overlap counts between DeepAdvancer-corrected and reference expression ratios for each class. **B**, Histogram showing the number of intersecting genes among the top 100 ranked by expression fold-change between each corrected dataset and the reference. Related to Figure 2.

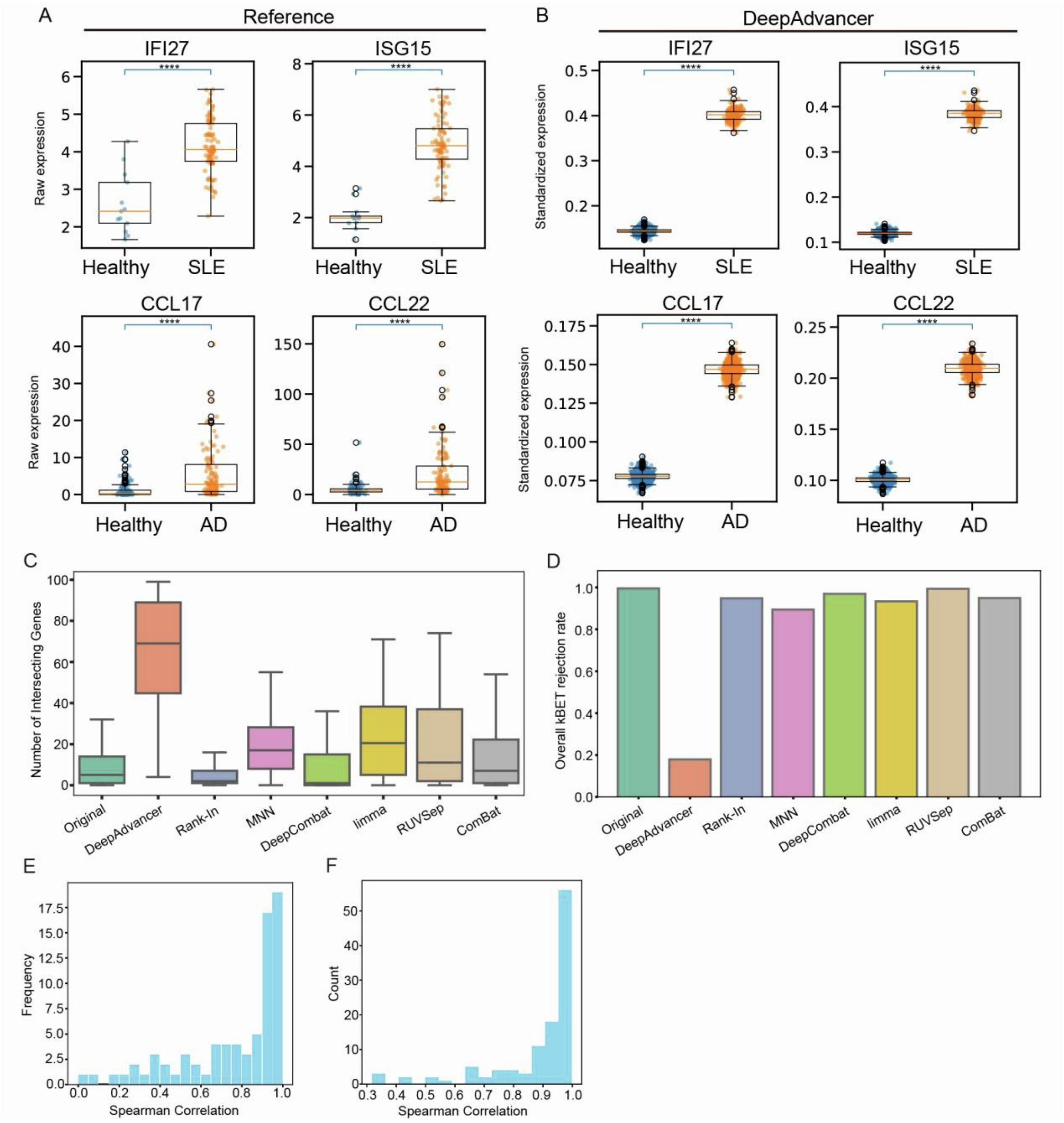


**Figure S2: Validation of disease-associated gene expression differences and robustness of batch correction and ratio reduction. A-B**, Identification of disease-associated gene expression differences. Comparison between the reference results (**A**) and DeepAdvancer (**B**) for representative genes across healthy and disease conditions (SLE, Systemic Lupus Erythematosus; AD, Atopic Dermatitis). Each dot represents one sample. Center lines indicate the median; box limits indicate the interquartile range (IQR); whiskers indicate 1.5 × IQR. Statistical significance was assessed using Student's t test. ****$p < 0.0001$. **C**, Comparison of intersecting gene counts across different batch correction methods. **D**, Quantitative evaluation of batch-effect removal using kBET. **E**, Distribution of spearman correlation coefficients between corrected and reference ratios for DeepAdvancer. **F**, Distribution of correlations after ratio reduction. Related to Figure 2.

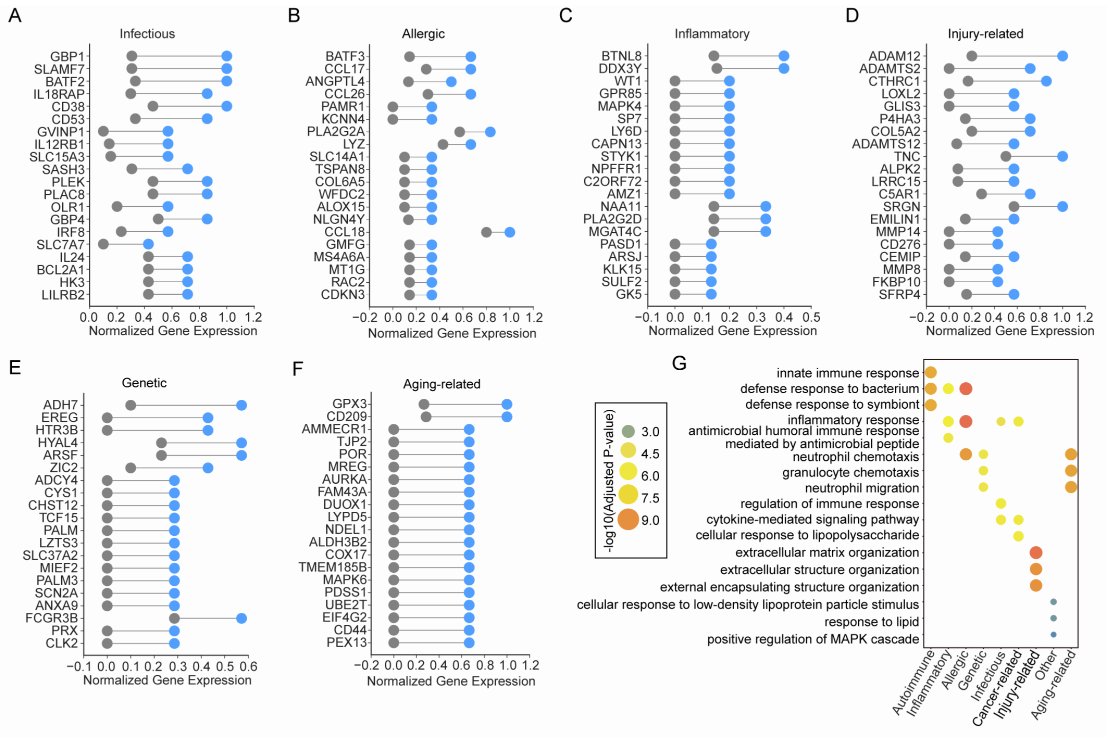


**Figure S3: Class-specific genes and marker pathways across disease classes. A-F,** The class-specific genes for each class**. G,** The marker pathways for each class. Related to Figure 3.

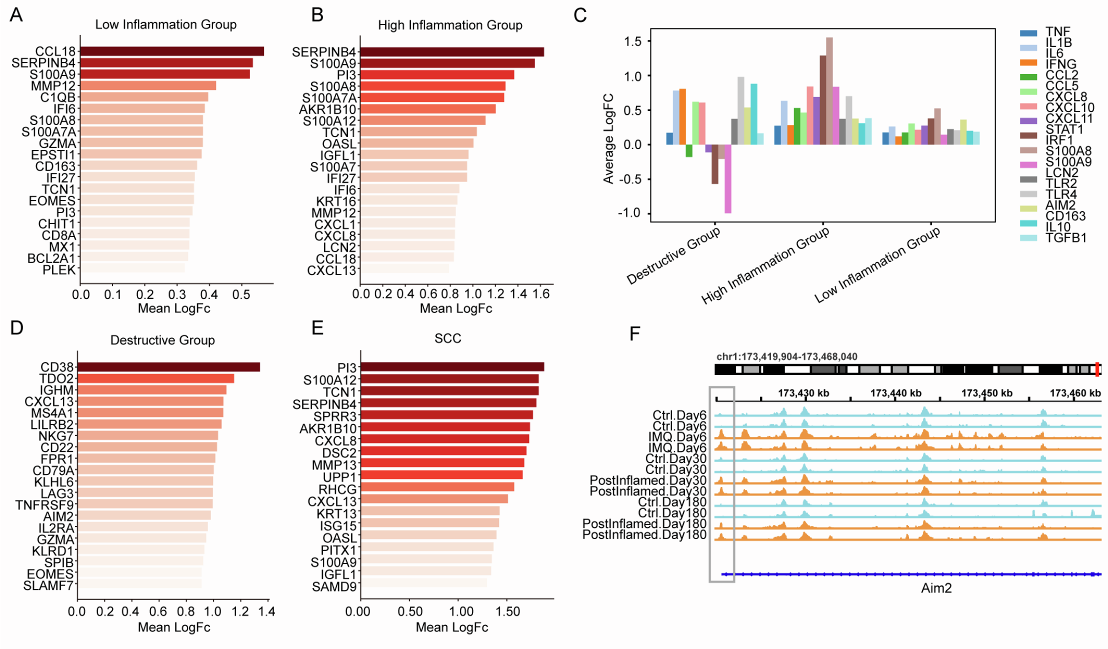


**Figure S4: Transcriptomic features across skin inflammation states. A–B**, **D-E**, Top genes with elevated expression in the low-inflammation group (**A**), high-inflammation group (**B**), tissue-destructive group (**D**), and squamous cell carcinoma (**E**). **C**, Inflammatory genes are upregulated in high-inflammation group. **F**, Snapshot of genomic loci whose chromatin-accessible peaks are opened by inflammation at D6 and persist up to 180D following resolution. Related to Figure 4.

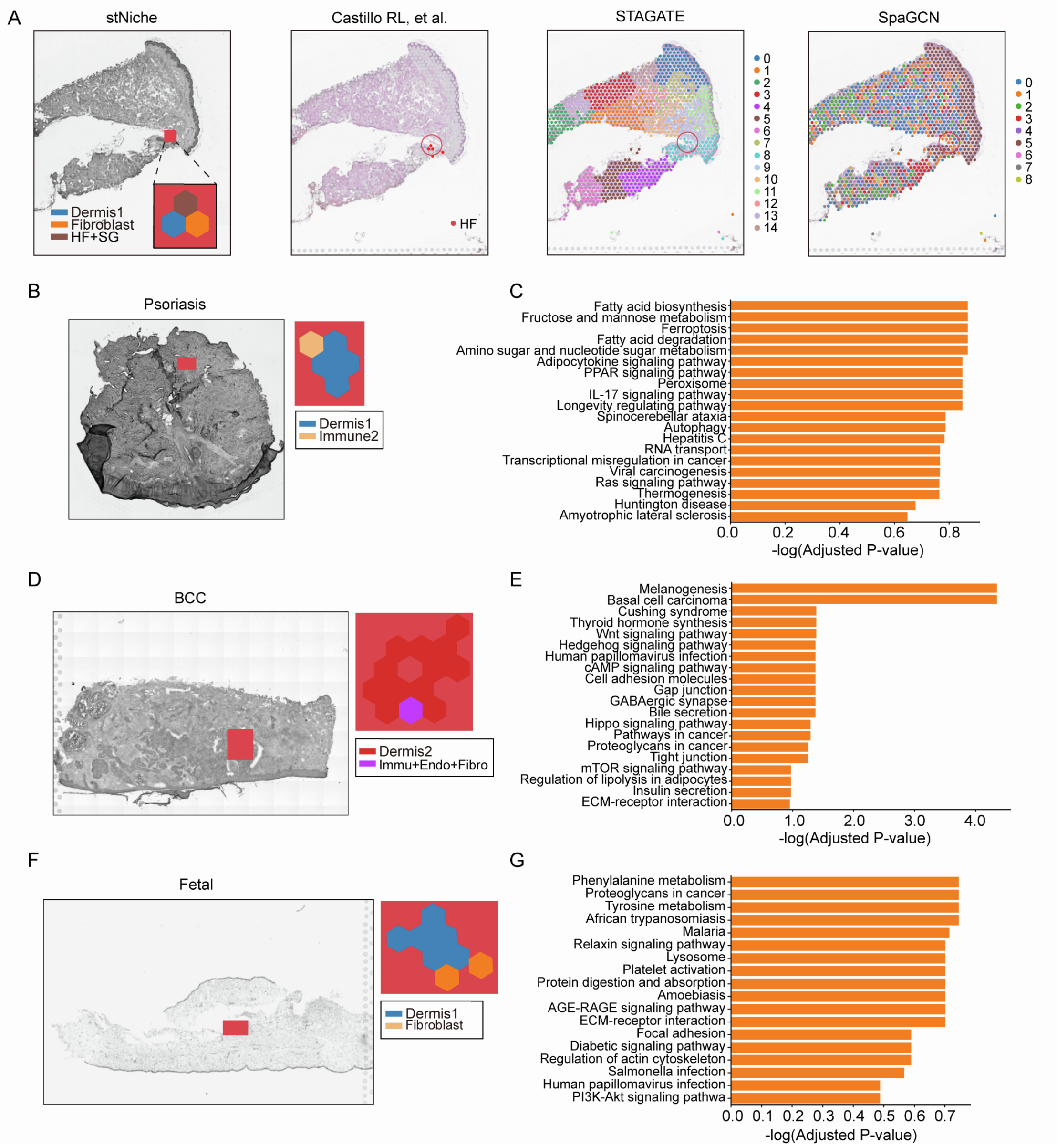


**Figure S5: Identification and functional profiling of spatial niches in various states. A**, Comparison of spatial niches identified by different methods. From left to right: the niche region identified by stNiche that distinguishes diseased from healthy states; the hair follicle (HF)-associated niche region reported in the original study; functional regions identified by STAGATE and SpaGCN. **B**, **D**, **F,** Spatial niches identified by stNiche in psoriasis (**B**), basal cell carcinoma (BCC) (**D**), and fetal skin tissue (**F**). **C**, **E**, **G**, Enriched functional pathways associated with the spatial niches in psoriasis (**C**), BCC (**E**), and fetal (**G**). Related to Figure 5.

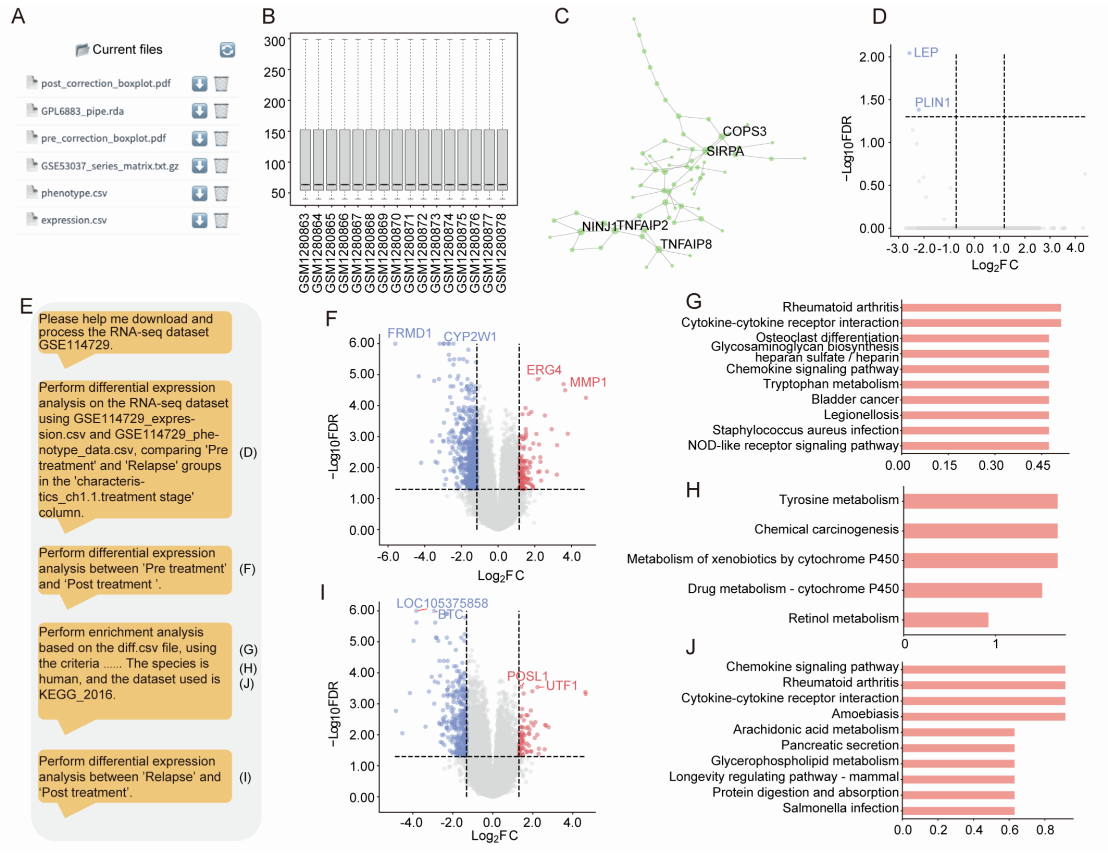


**Figure S6: BioinAI-Web supports automated analysis of bulk RNA-seq data. A**, Illustration of BioinAI-Web showing result download functions. **B**, Preprocessed microarray dataset visualized after normalization. **C**, Module 3 derived from WGCNA analysis on differentially expressed genes from the microarray data. **D, F**, **I**, Volcano plots showing differentially expressed genes in comparisons between relapse and post-treatment (**D**), pre-treatment and post-treatment (**F**), and post-treatment and relapse (**I**) samples. **E**, Sequence of user prompts to analyze a bulk RNA-seq dataset and the corresponding results generated by the online platform. **G**-**H**, **J**, Bar plots of enriched functional terms for genes upregulated in post-treatment vs. pre-treatment (**G**), downregulated in post-treatment vs. pre-treatment (**H**), and upregulated in relapse vs. post-treatment (**J**). Related to Figure 6.

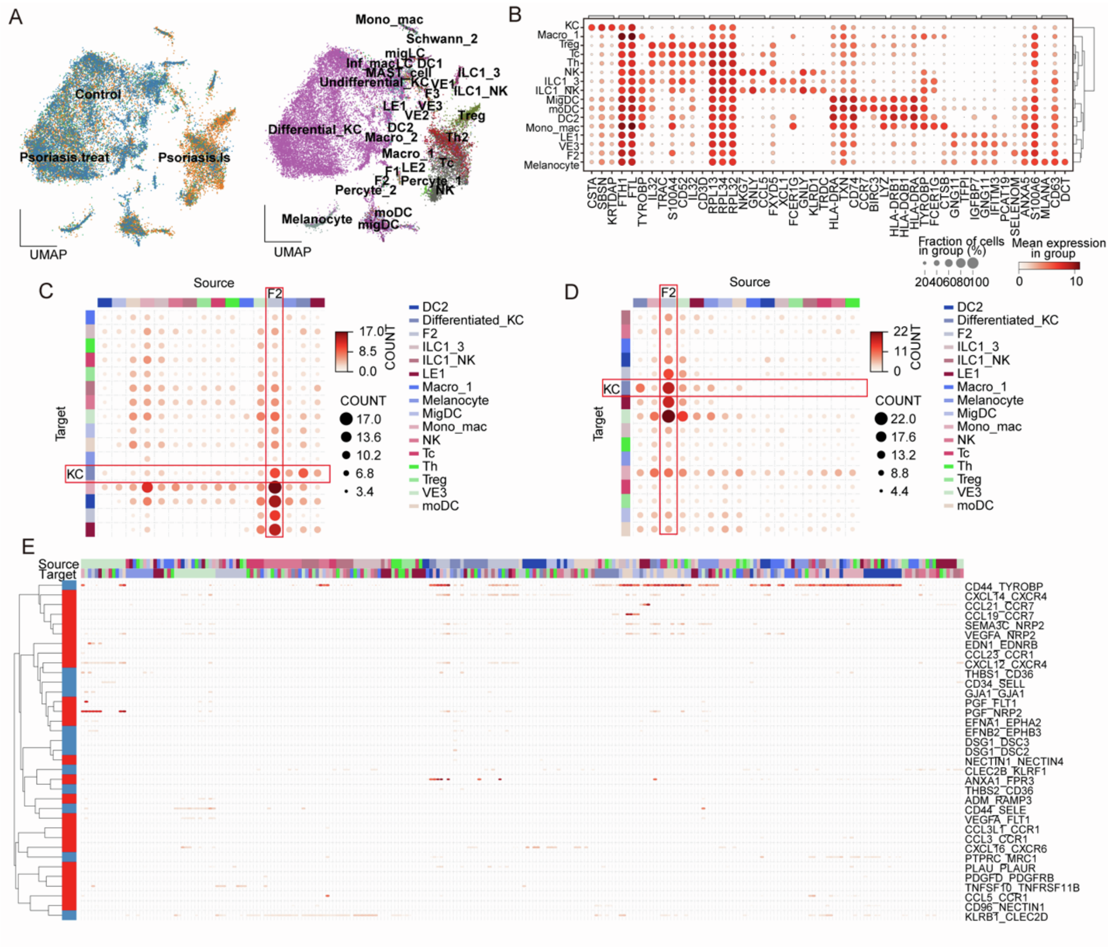


**Figure S7: BioinAI-Web enables analysis of single-cell transcriptomic data. A**, UMAP plots of integrated single-cell RNA-seq data colored by group (left) and refined cell-type annotations (right). **B**, Dot plot showing expression of marker genes across subpopulations. **C**, **D**, Heatmaps illustrating intercellular communication among subpopulations in healthy controls (**C**) and post-treatment samples (**D**). **E**, Summary of signaling pathways. Related to Figure 7.

**Table S2. Computational cost of model training across different sample sizes**

| Samples | Train time (s) | Train time (min) | Peak allocated (GB) | Peak reserved (GB) |
|---|---|---|---|---|
| 1000 | 133.95 | 2.23 | 0.913 | 1.428 |
| 5000 | 674.42 | 11.24 | 2.702 | 3.668 |
| 10000 | 1345.19 | 22.42 | 4.94 | 6.463 |
| 20000 | 2691.47 | 44.86 | 9.41 | 11.86 |

**Table S3. Summary of skin-related spatial transcriptomics datasets included in this study**

| Dataset ID | Database Source | Disease Category | Number of Samples |
|---|---|---|---|
| E-MTAB-13024 | ArrayExpress | Fetal skin | 4 |
| E-MTAB-13084 | ArrayExpress | Basal cell carcinoma | 30 |
| E-MTAB-13614 | ArrayExpress | Cutaneous T-cell lymphoma | 8 |
| E-MTAB-14559 | ArrayExpress | Healthy skin | 15 |
| GSE144239 | GEO | Squamous cell carcinoma | 4 |
| GSE173651 | GEO | Healthy skin | 6 |
| GSE197023 | GEO | Atopic dermatitis | 19 |
| GSE202011 | GEO | Psoriasis | 30 |
| GSE206391 | GEO | Inflammatory skin diseases | 66 |
| GSE225475 | GEO | Psoriasis | 6 |
| GSE241124 | GEO | Wound healing / wound skin | 16 |
| GSE263298 | GEO | Cutaneous leishmaniasis | 12 |
| GSE263571 | GEO | Granuloma annulare | 12 |
| tildra | Zenodo | Psoriasis | 12 |
